# The Efficacy of Fecal Microbiota Transplantation for Children With Tourette Syndrome: A Preliminary Study

**DOI:** 10.3389/fpsyt.2020.554441

**Published:** 2020-12-23

**Authors:** Hui-Jun Zhao, Xi Luo, Yi-Chao Shi, Jian-Feng Li, Fei Pan, Rong-Rong Ren, Li-Hua Peng, Xiu-Yu Shi, Guang Yang, Jing Wang, Lin-Yan Hu, Li-Ping Zou, Yun-Sheng Yang

**Affiliations:** ^1^Department of Gastroenterology and Hepatology, The First Medical Center, Chinese People's Liberation Army General Hospital, Beijing, China; ^2^Department of Pediatrics, The First Medical Center, Chinese People's Liberation Army General Hospital, Beijing, China

**Keywords:** tourette syndrome, fecal microbiota transplantation, shotgun metagenomics, lipopolysaccharide, cytokines

## Abstract

Therapies for Tourette syndrome (TS) are insufficient, and novel therapies are needed. Fecal microbiota transplantation (FMT) has been a potential therapy for several neurological diseases. Here, we report a preliminary study to investigate the effects of FMT on patients with TS. Five patients with TS received a single administration of FMT via endoscopy. Tic symptoms were assessed by Yale Global Tic Severity Scale-Total Tic Score (YGTSS-TTS) and adverse effects were recorded at week 8 following FMT. Lipopolysaccharide (LPS) levels and 14 cytokines levels were measured. The microbiota profile in feces were analyzed by shotgun metagenomics. Four patients (4/5) responded positively to FMT (YGTSS-TTS reduction rate >25%) at week 8 with high safety. The levels of LPS and cytokines varied after FMT. FMT shifted the composition of the gut microbiota in patients close to that of the donor and continuously changed the abundance of *Bacteroides coprocola, Dialister succinatiphilus* and *Bacteroides vulgatus*. The restoration of *B.coprocola* was correlated with the improvement in tic symptoms (Spearman *R* = −0.900, *P* = 0.037). In conclusion, FMT was indicated a potential effective and safe alternative for patients with TS. However, larger clinical trials are needed to confirm the influence of microbiota in TS.

**Trial Registration:** chictr.org.cn Identifier: ChiCTR-IIR-17011871, URL: http://www.chictr.org.cn/showproj.aspx?proj=19941.

## Introduction

Tourette syndrome (TS) is a combination of persistent multiple motor tics and at least one kind of vocal tic lasting for more than 1 year in youths before reaching the age of 18 years old (1). The prevalence of TS is ~0.8% worldwide and 1.7% in China (2, 3). Approximately 80–90% of patients with TS have common neuropsychiatric comorbidities, such as attention-deficit/hyperactivity disorder (ADHD), obsessive-compulsive disorder (OCD), anxiety, depressive disorders, and autism spectrum disorders (ASDs) (4–6). Generally, genetic and environmental factors play a substantial role in the onset of TS; however, the intrinsic etiologies are currently poorly understood. Environmental factors include pre- and perinatal factors (7), psychosocial stress (8), and abnormal innate and adaptive immune responses (9). In addition, increasing evidence indicates that infections and immune activation might be part of the pathogenesis of TS (10). Infections with group A streptococci (GAS) have been implicated in the development of pediatric autoimmune neuropsychiatric disorders associated with streptococcal infections (PANDAS), which is a subtype of pediatric OCD and/or TS. One potential mechanism of PANDAS is that GAS induce inflammatory/immunological dysregulation (11). Abnormal activation of the Toll-like receptor (TLR) pathway induced by lipopolysaccharide (LPS) produced by gram-negative bacteria has been observed in TS patients. LPS has further been found to aggravate tic symptoms and increase inflammatory cytokine production in rat models of TS (12).

Behavioral therapy and pharmacotherapy for TS are conducted with an emphasis on the individual. Pharmacotherapy includes antipsychotic medications (haloperidol, tiapride, haloperidol, and risperidone and aripiprazole) and alpha agonists (clonidine and guanfacine) (6). However, a certain proportion of patients may fail to respond to pharmacological treatments alone or in combination, and the above medications produce some potential adverse effects, such as drug-induced movement disorders, metabolic and hormonal effects, and sedation (13). Deep brain stimulation (DBS) has been used extensively as an invasive neuromodulation method in patients with severe, medically refractory TS. However, uncertainties surrounding targeted anatomical selection, controversial age cut-offs for patient consideration for surgery and a high frequency of postoperative infections are drawbacks of DBS (13). The TS requires further research to clarify its pathogenesis, and alternative treatment options still need to be explored.

Previous studies have suggested that gut microbiota substantially influence the development of the brain and behavior through bidirectional communication via the microbiota-gut-brain axis (14). ASD and ADHD, which are neurological diseases and sometimes co-occur with TS, has been associated with altered gut microbial profiles: decreased Alistipes, Dialister, Veillonella and increased Collinsella, Dorea, and Lactobacillus abundances are found in ASD patients (15); decreased Faecalibacterium abundance has been found in ADHD patients (16).

There are several interventions for modulating gut microbiota to relieve behavioral abnormalities, including probiotics, antibiotics, diet, and especially fecal microbiota transplantation (FMT) (14). FMT, which reconstitutes the balance of patients gut microbiota with that of fecal microbiota from healthy donors, has been effectively used for treating recurrent Clostridium difficile infection (17) and has also been used to treat inflammatory bowel disease (18) and hepatic encephalopathy (19). Recent clinical trials have further shown that FMT could persist in alleviating the symptoms of ASD (20) and epilepsy (21) by reconstituting the recipient gut microbiota. In addition, increasing evidence shows that gut microbiota modulates different neurological diseases via different mechanisms of the microbiota-gut-brain axis. For example, a previous study showed that TLR-4-mediated inflammation triggers intestinal and/or brain inflammation, which further aggravates neurodegeneration in Parkinson's disease patients (22); the gut microbiome of patients with schizophrenia alters the glutamate-glutamine-GABA cycle and worsens schizophrenia-relevant behaviors (23); microbial reconstitution reverses the social and synaptic deficits of maternal high-fat diet (MHFD) offspring by correcting oxytocin levels and synaptic potentiation (LTP) in the ventral tegmental areas (VTAs) (24).

Considering that new alternatives are urgently required to relieve the symptoms of patients with TS who fail to respond to medications or DBS, and the changes in gut microbial populations are correlated with multiple neuropsychiatric diseases (20–23), it is worth exploring the effect of FMT in TS. Our research team previously focused on performing FMT to patients with ulcerative colitis (UC), and achieved high clinical response and safety (25). Then we conducted an FMT to one patient with TS and found that the tic symptoms assessed by the Yale Global Tic Severity Scale-Total Tic Score (YGTSS-TTS) were ameliorated notably by 8 weeks (26). Here, we performed FMT treatment on five patients with TS to assess its efficacy and safety; we further explored the alterations of fecal microbial composition and serum cytokines after FMT.

## Materials and Methods

### Ethical Approval

This trial was approved by the Ethical Committee of Chinese People's Liberation Army General Hospital (S2015-110-02) and registered in the Chinese Clinical Trial Registry (ChiCTR-IIR-17011871). The patients and their legal representative and/or the patients voluntarily participated in the study and signed the informed consent form.

### Patient Recruitment

Five patients meeting the diagnostic criteria of the Diagnostic and Statistical Manual of Mental Disorders, Fifth Edition (DSM-V) and YGTSS-TTS (combined motor tic and vocal tic score) > 13 were included (27). Patients were eligible if they had been diagnosed for more than one year, had a persistent high level of tic severity, and had a relapse or were intolerant to regular medications for tics disorders. Patients continued to receive regular medication therapy for tics if the medication was stable for at least 3 weeks and no changes occurred over the 8-week trial. Enrolled patients were required to stop using antimicrobial drugs or probiotics for more than 1 month.

### Donor Screening and FMT Procedure

A questionnaire about the family medical history and individual medical history, and laboratory blood and fecal examinations for pathogens were used for donor screening. Healthy fecal donor inclusion criteria were as follows: aged 5–30 years without sex limitation. Exclusion criteria included known infectious diseases, current gastrointestinal disease, and other systematic diseases, and use of medications causing dysbiosis. One hundred and thirty one volunteers were screened for eligibility, including 59 males and 72 females, with an average age of 33.5 (range 3–67) years. Five people were enrolled as donors according the inclusion and exclusion criteria. In a separate study, we performed FMT to 62 patients with UC from the five donors included in the current study and compared the rate of clinical remission (defined as a total Mayo score (for UC activity) ≤ 2, combined with all Mayo subscores ≤ 1). We subsequently identified that the efficacy of FMT using the feces from a 14-year-old male was superior to those using other donors after comparison (data unpublished). Therefore, we selected the 14-year-old male donor for the five TS patients in the current study. We collected ~120 g of fresh stool for every treatment. The stool was homogenized with 500 ml physiological saline (Kelidai, China) and then filtered to an ~400 ml suspension (donor fecal liquid, DL). After intravenous anesthesia, FMT treatment was performed with 100 ml of fecal suspension delivered through a gastroscope into the duodenum and 300 ml delivered to the colon via a colonoscopy. The detailed process of donor screening and the FMT procedure were previously reported in Wang et al. (25).

### Outcomes

**Figure 1:**
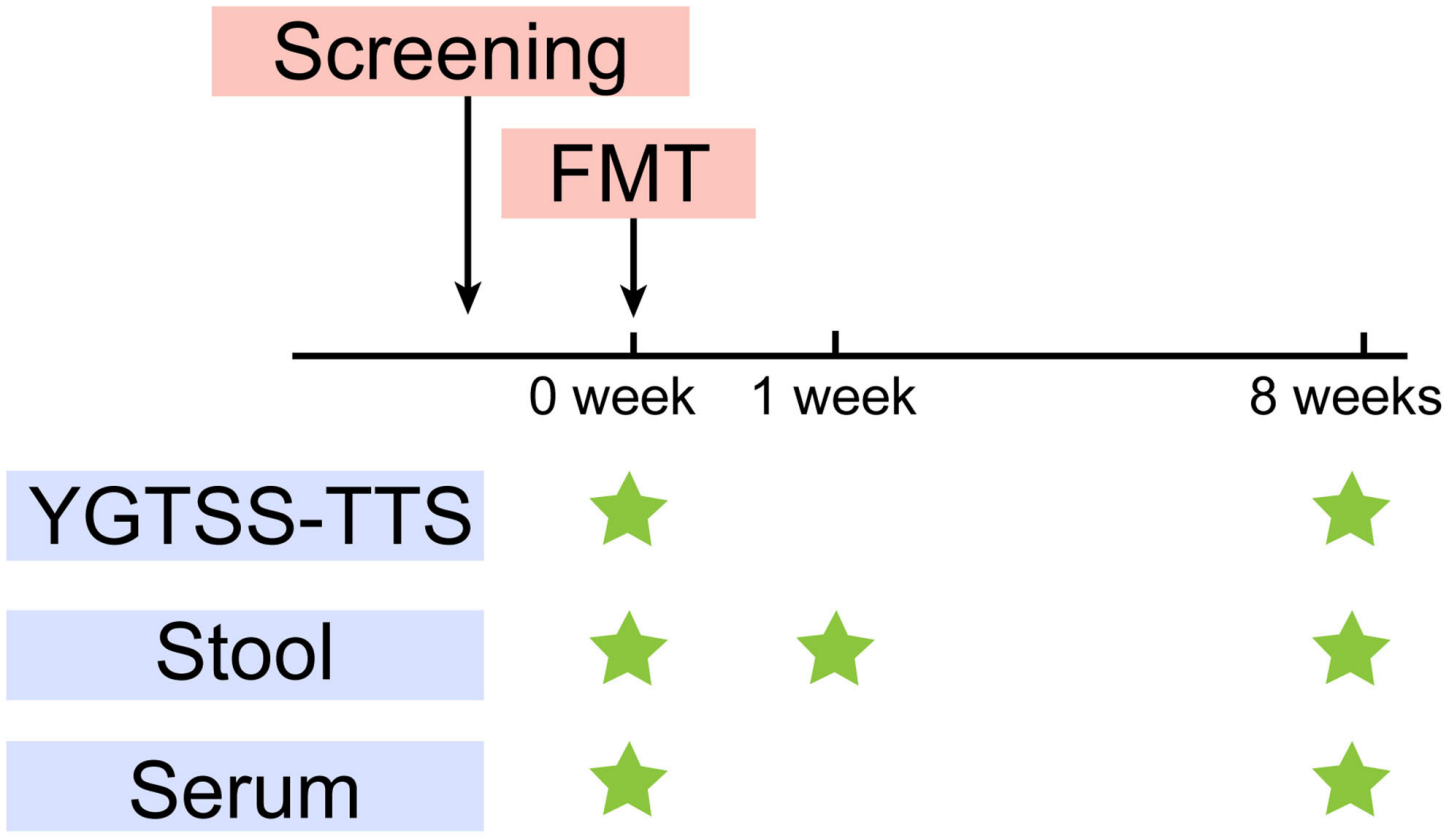
Study design. After screening, FMT was performed at W0. YGTSS-TTS was assessed at baseline (W0) before FMT and W8. Stool samples were collected for metagenome sequencing and lipopolysaccharide (LPS) measurement at W0, W1, W8. Serum samples were collected for LPS and cytokines measurement at W0, W8.

The primary outcome was the YGTSS-TTS. The YGTSS is a multi-dimensional, clinician-rated scale assessing tic severity, including the Total Tic Score (TTS) (0–50) and the Overall Impairment Score (0–50). The YGTSS-TTS is the combination of the Total Motor Tic score and the Total Vocal Tic score, which are assessed separately from five dimensions: the number, frequency, intensity, complexity and inference. Each item is scored from 0 to 5. Clinical response is defined as a YGTSS-TTS score-reduction rate of > 25% (27). We assessed the tic severity at baseline (W0) and 8 weeks after treatment (W8) using YGTSS-TTS (Figure 1), which was conducted by an independent evaluator.

### Sample Collection

The fecal samples were collected from the five TS patients at W0, W1 (1 week after FMT) and W8. The donor feces (DF) was collected for every treatment. We further collected the DL, which represented the microbial composition that was ultimately transplanted into the patients. We also collected serum samples from patients at W0 and W8 (Figure 1). All the samples were stored at −80°C.

### Limulus Amoebocyte Lysate Assay and Cytokine Analysis

We detected fecal and serum LPS levels and the serum levels of 14 cytokines. Serum and fecal LPS levels were measured using a limulus amoebocyte lysate (LAL) assay (Xiamen Bioendo Technology Co., Ltd, Xiamen, China) following the manufacturer's protocol. In brief, serum was diluted 10-fold in pyrogen-free water and inactivated at 70°C for 10 min. One gram of fecal sample was dissolved in 10 ml of sterile PBS, vortexed gently, and centrifuged at 3,000 rpm for 15 min. The supernatant was filtered through a 0.45-μm filter and a 0.22-μm filter successively and inactivated at 90°C for 15 min (28). The cytokine analysis was conducted using a multiplexing bead immunoassay (AimPlex Biosciences, Inc., USA) following the manufacturer's protocol to measure the levels of 14 different cytokines: IL-1β, IL-2, IL-4, IL-5, IL-6, IL-8, IL-10, IL-12P70, IL-17A, IL-17F, IL-22, TNFα, TNFβ, and IFN-γ. Briefly, 45 μl samples were incubated with antibody-coupled fluorescent beads, washed and incubated with Biotin-dAb and NR-dAb diluent. The beads were analyzed using a flow cytometer (BD Bioscience, San Diego, CA, USA). Standard curves were generated by FCAP Array 3.0 software (BD Bioscience, San Diego, CA, USA) to determine the concentration of unknown sample.

### Shotgun Metagenomic Sequencing and Analysis

Shotgun metagenomic sequencing was conducted as previously reported (29). In brief, DNA in the fecal sample (200 mg) and DL (200 μl) was extracted using a QIAamp DNA Stool Mini kit (QIAGEN), and the concentration was gauged by a NanoDrop instrument. The DNA library construction was performed following the Illumina TruSeq DNA Sample Prep v2 Guide, and the libraries were sequenced using an Illumina HiSeq 4000 (10G per sample). After data quality control and host genome filtering, the Illumina short reads were *de novo* assembled using SOAP *de novo* software (V2.04), and the obtained Scaffolds were cut into contigs, which were further used for gene prediction. The microbial composition at different taxonomic levels was annotated by MEGAN software (version 5, http://ab.inf.uni-tuebingen.de/data/software/megan5/download/welcome.html) with matched genes. The abundance of a taxonomic group equalled the sum of the gene abundance annotated to the species. The genes were assigned to the Kyoto Encyclopedia of Genes and Genomes (KEGG) orthology/module group for functional annotation.

### Statistical Analysis

Non-normally distributed continuous data were presented as median (range). LPS and cytokines levels were analyzed by Wilcoxon signed-rank test. The principal component analysis (PCA) was calculated by the unweighted UniFrac distance metric and the analysis of similarities (ANOSIM) by the Vegan package in R (version 2.15.3). Microbiota differential abundance and function comparisons were performed using the linear discriminant analysis effect size (LEfSe) algorithm (http://huttenhower.sph.harvard.edu/galaxy), and an LDA score >3 was applied. Further pairwise comparisons of relative abundance among groups were analyzed via Metastats (http://metastats.cbcb.umd.edu/) (30). Spearman's correlation coefficient was calculated using R. *P* < 0.05 was considered statistically significant.

## Data Availability

The metagenomic sequencing data are available in the BioProject database under research ID PRJNA628029 and the SRA accession number SUB7328137 (https://submit.ncbi.nlm.nih.gov/subs/sra/SUB7328137).

## Results

### Patient Characteristics

Five boys with TS were enrolled for FMT treatment in this pilot study (Table 1). The average age of the patients was 8 years (range 7–10 years), and the body mass index (BMI) was 18.0 (range 13.2–26.3). The disease duration before FMT ranged from 1.5 to 4 years. All their anti-streptolysin O titres (ASOT) tests were negative. Patients 2,3, and 4 had TS combined with ADHD; patient 3 had variant asthma.

**Table 1:** The general information of the five patients.

**Code**	**Sex**	**Age (years)**	**BMI**	**Duration (years)**	**ASOT**	**Comorbidities**	**Concomitant medication**
P1	M	7	13.2	4	_	NR	Tiapride
P2	M	10	26.3	4	_	ADHD	Aripiprazole, trihexyphenidyl
P3	M	10	20.7	3	_	ADHD, Variant asthma	Aripiprazole, risperidone, trihexyphenidyl
P4	M	9	19.5	4	_	ADHD	Tiapride
P5	M	7	16.4	1.5	_	NR	Tiapride

The symptoms of tic disorders in patients persisted for more than 1 year, and concomitant medications were used during the treatment.

*BMI, body mass index; ASOT, anti-streptolysin O titres; M, male; ADHD, attention-deficit/hyperactivity disorder; NR, no report*.

### Efficacy and Safety of FMT in TS Patients

Table 2 show the YGTSS-TTS results for five patients. Four patients (4/5) achieved a clinical response at week 8 after FMT; patient 2 was the exception. The YGTSS-TTS of the remaining four patients decreased with a range of 7–35. The total tic symptoms of patient 3, who failed to adequately respond to medication, disappeared at week 8, and the vocal tic symptoms of patient 1 were also completely resolved. However, the vocal tic score of patient 2 increased from 13 to 17, which indicated a slight aggravation of symptoms. During the FMT process and follow-up period, no patients experienced any obvious adverse events, such as allergy, nausea, vomiting, diarrhea or abdominal pain. The body temperature of all five patients rose slightly 24 h after FMT treatment (Table 3).

**Table 2:** YGTSS-TTS scores and reduction rate in the five patients.

	**Motor tic score**			**Vocal tic score**			**Total tic score**			
	**W0**	**W8**	**Change**	**W0**	**W8**	**Change**	**W0**	**W8**	**Change**	**Reduction rate %**
P1	20	7	−13	16	0	−16	36	7	−29	80.6
P2	14	14	0	13	17	4	27	31	4	−14.8
P3	18	0	−18	17	0	−17	35	0	−35	100
P4	11	9	−2	10	5	−5	21	14	−7	33.3
P5	24	8	−16	12	4	−8	36	12	−24	66.6

W0, Baseline; W8, Eight weeks after treatment.

*DF, donor feces; DL, donor fecal liquid; W0, baseline of patients; W1, week 1 after FMT; W8, week 8 after FMT*.

**Table 3:** The adverse events after FMT.

	**Temperature (**^****°****^**C)**		**Allergy**	**Nausea**	**Vomiting**	**Diarrhea**	**Abdominal pain**
	**Pre-FMT**	**Post-FMT**					
P1	36.8	37.2	-	-	-	-	-
P2	36.1	37.1	-	-	-	-	-
P3	36.5	37.0	-	-	-	-	-
P4	37.2	37.2	-	-	-	-	-
P5	36.8	37.2	-	-	-	-	-

*The body temperature of all five patients rose slightly 24 h after FMT treatment*.

### Variations in LPS and Cytokine Levels Following FMT

**Figure 2:**
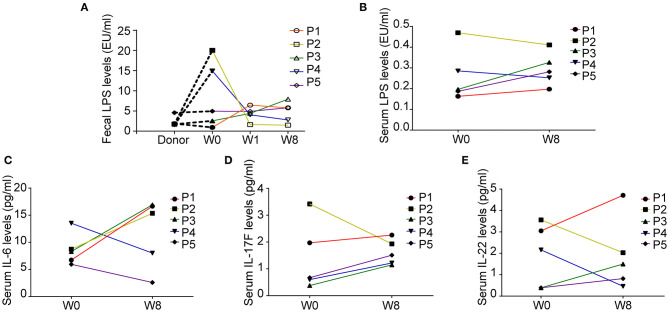
Fecal and serum lipopolysaccharide (LPS) levels and serum cytokine levels in patients with Tourette Syndrome (TS) following FMT. **(A)** Fecal LPS levels of the donor and five patients with TS. **(B)** Serum LPS levels of five patients with TS before and after FMT. **(C)** Serum IL-6 levels. **(D)** Serum IL-17F levels. **(E)** Serum IL-22 levels.

We explored the changes in LPS and 14 cytokines in TS patients. There were no significant changes in the LPS concentration in the feces and serum or in the concentration of all the cytokines in the serum before and after FMT (Table 4). The LPS concentrations of patients 2 and 4 in stool and serum samples were dramatically higher than those of the donor at baseline and persistently decreased after FMT (Figures 2A,B). The concentration of serum IL-6 was reduced in patient 4 and patient 5 following FMT but elevated in the other three patients (Figure 2C). Levels of both IL-17F and IL-22 were decreased in patient 2 (Figures 2D,E).

**Table 4:** Fourteen cytokine levels in the serum before and after FMT.

	**W0 (pg/ml), median(range)**	**W8 (pg/ml), median(range)**	**z**	***P***
IL-1β	1.52 (1.30–2.27)	1.68 (0.79–2.41)	−0.674	0.500
IL-2	2.30 (0.84–4.91)	2.45 (0.90–43.43)	−0.405	0.686
IL-4	1.64 (0.41–3.77)	0.44 (0.10–1.16)	−1.753	0.080
IL-5	1.05 (0.51–3.91)	0.90 (0.80–1.66)	−0.542	0.588
IL-6	8.30 (5.95–13.53)	15.36 (2.59–16.92)	−1.214	0.225
IL-8	7.05 (2.19–34.00)	10.06 (3.85–57.18)	−0.405	0.686
IL-10	3.61 (2.66–4.66)	5.01 (3.24–28.80)	−1.753	0.080
IL-12P70	3.31 (2.61–4.00)	3.32 (3.08–3.83)	−0.135	0.893
IL-17A	0.95 (0.44–3.87)	1.17 (0.20–2.06)	−0.135	0.893
IL-17F	0.67 (0.38–3.42)	1.51 (1.15–2.26)	−0.674	0.500
IL-22	2.16 (0.39–3.56)	1.50 (0.46–4.71)	−0.135	0.893
TNFα	3.59 (3.08–4.30)	3.78 (2.81–4.36)	−0.944	0.345
TNFβ	1.84 (1.39–1.96)	1.94 (1.67–2.16)	−1.214	0.225
IFNγ	3.07 (2.68–4.12)	3.31 (2.25–5.13)	−0.135	0.893

### Alterations in Patient Microbiota Composition Before and After FMT

**Figure 3:**
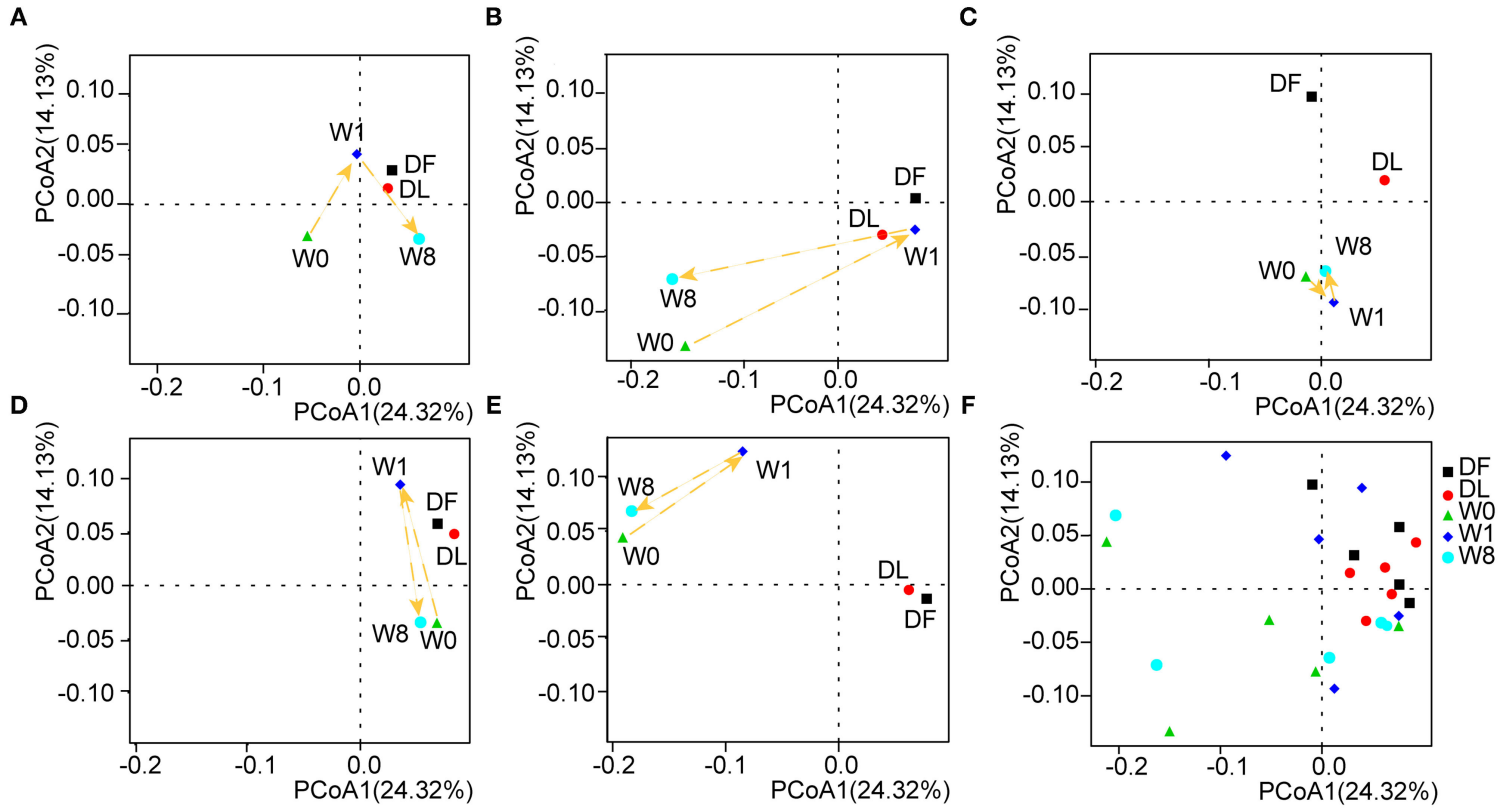
The shift in gut microbiota in the five patients before and after FMT. Principal coordinates analyses (PCoA) was performed by the unweighted UniFrac distance metric using the whole data set and showed a shift in the gut microbiota of each patient separately **(A–E)** and in combination **(F)**.

We further investigated the microbiota composition and diversity in TS patients before and after FMT by metagenomics analysis. The number of non-redundant genes in TS patients prior to FMT was significantly decreased compared to that in the donor (*P* = 0.073, DF VS W0; *P* = 0.043, DL VS W0, analysis of variance (ANOVA), Supplementary Figure 1) and transiently increased following FMT treatment (*P* = 0.053, W0 VS W1, ANOVA, Supplementary Figure 1). Principal coordinates analyses (PCoA) calculated by the unweighted UniFrac distance metric revealed a clear cluster of patients with TS prior to FMT away from the donor (Figure 3F). The gut microbiota composition in TS patients 1, 2, 4, and 5 temporarily shifted close to the profile of the donor microbiota by W1 but moved away by W8 though remaining dissimilar to the composition prior to FMT and to that of the healthy donor (Figures 3A,B,D,E). However, there was no obvious change observed in patient 3 whose microbiota composition remained distinct from the donor (Figure 3C). The analysis of similarity (ANOSIM) showed a similar trend in microbial composition among the different time points (DF VS W0: R = 0.268, *P* = 0.006; DF VS W1: *R* = 0.092, *P* = 0.161; DF VS W8: *R* = 0.212, *P* = 0.036; W0 VS W1: *R* = 0.092, *P* = 0.235; W0 VS W8: *R* = −0.08, *P* = 0.714; Supplementary Figure 2), indicating that FMT treatment transiently altered the gut microbiota composition of patients to that of the donor.

**Figure 4:**
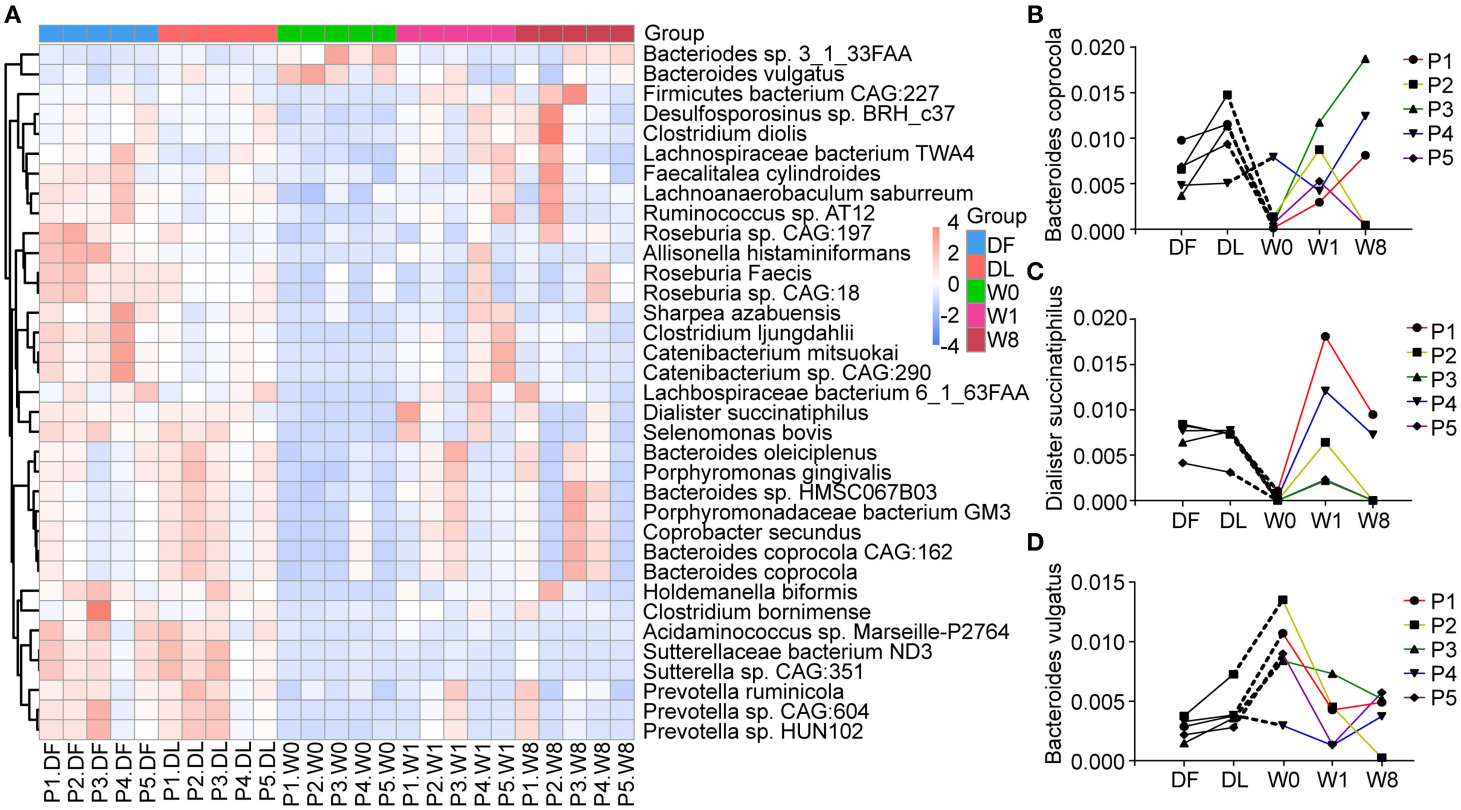
Changes in microbial composition at the species levels after FMT generated with Metastats. **(A)** A heatmap showing the relative abundance of 35 species in different groups. **(B–D)** The dynamic changes in *Bacteroides coprocola, Dialister succinatiphilus*, and *Bacteroides vulgatus* in Tourette Syndrome (TS) patients.

We further explored changes in the microbiota composition of TS patients following FMT. Four different phyla, Firmicutes (53%), Bacteroidetes (27%), Actinobacteria (4%) and Proteobacteria (4%), dominated the gut microbiota composition of the five TS patients (Supplementary Figure 3). We performed LEfSe analysis and Metastat analysis to investigate the significant differences between TS patients and the healthy donor. LEfSe analysis revealed that the genera Bifidobacterium, Collinella, Dorea and Catenibacterium were much lower in TS patients than in the donor, and the abundance of the above genera transiently increased to the level of the donor at W1 but decreased by W8 (Supplementary Figure 4). Furthermore, the abundance of species *Roseburia faecis, Bacteroides coprocola, Dialister succinatiphilus, Catenibacterium mitsuokai, Holdemanella biformis*, and *Allisonella histaminiformans* was significantly decreased in TS patients compared with the healthy donor (relative abundance of DF VS W0, 0.0153 VS 0.0032, *P* = 0.0001; 0.0064 VS 0.0021, *P* = 0.0161; 0.0070 VS 0.0003, *P* = 1.59^*^10^−6^; 0.0051 VS 0.0002, *P* = 0.0009; 0.0075 VS 4.8^*^10^−5^, *P* = 0.0004; 0.0016 VS 2.8^*^10^−5^, *P* = 0.0002, Figure 4A); however, the abundance of *Bacteroides vulgatus* was significantly increased (relative abundance of DF VS W8, 0.0027 VS 0.0089, *P* =0.0017) (Figure 4A). Following FMT, *Bacteroides coprocola* abundance was restored in patients 3, 4, and 5 (Figure 4B), and the abundance of *Dialister succinatiphilus* was restored in patients 4 and 5 (Figure 4C). However, *Bacteroides vulgatus* abundance remained continuously reduced in all five patients (relative abundance: W0 = 0.0089, W1 = 0.0038, W8 = 0.0040, P_*W*0*vsW*1_ = 0.0372, P _*W*0*vsW*8_ = 0.0380) (Figure 4D). Furthermore, the change in YGTSS-TTS showed a strong negative correlation with the abundance of *Bacteroides coprocola* (Spearman *R* = −0.900, *P* = 0.037).

### Functional Transformation/Diversification Following FMT Treatment

**Figure 5:**
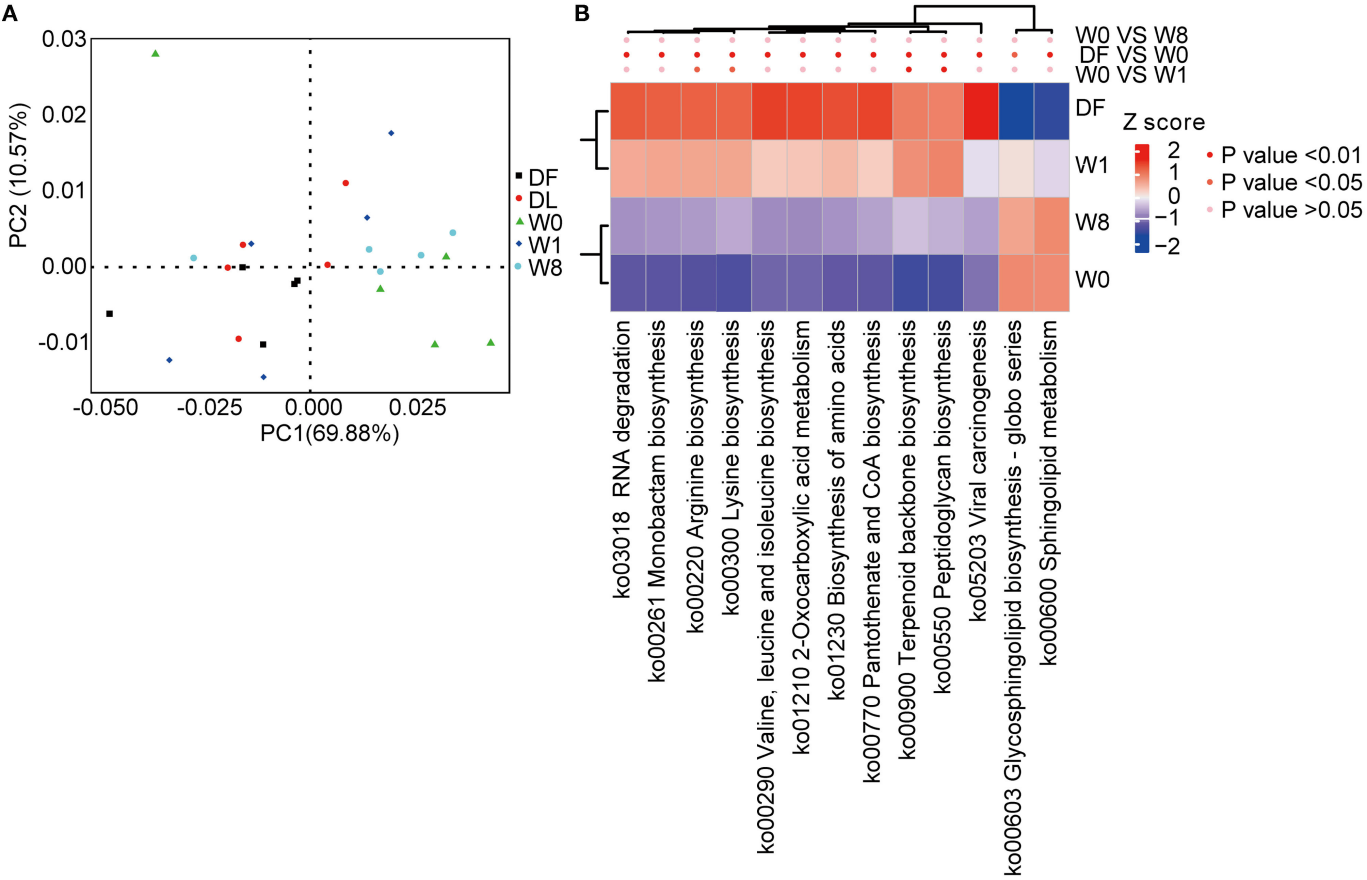
Microbial functional differences after FMT assessed by metastats. **(A)** principal component analysis (PCA) showing the dynamic alterations in functional features. **(B)** A heatmap showing the significant differences in several Kyoto Encyclopedia of Genes and Genomes (KEGG) pathways.

We characterized the functional changes in the gut microbiota using the KEGG database to annotate the metagenomics data. Dramatic functional differences between TS patients and healthy donors and post-FMT changes in TS patients resembling the donor gut microbiota were found (Figure 5A). The genes predominantly related to the biosynthesis of amino acids, glycan biosynthesis, and metabolism were significantly different between TS patients (W0) and healthy donors (DF). Amino acid biosynthesis contributing to arginine biosynthesis, lysine biosynthesis, terpenoid backbone biosynthesis and peptidoglycan biosynthesis pathways were significantly enriched, whereas glycosphingolipid biosynthesis and sphingolipid metabolism pathways were depleted promptly after FMT treatment (W1); however, all KEGG KOs had shifted back to the primary state by W8 (Figure 5B).

## Discussion

Herein, we report the significant clinical efficacy and safety of FMT for treating patients with TS and assessed for changes in LPS, cytokine levels, and microbial composition following the modulation of gut microbiota through FMT.

FMT has been shown to have a certain therapeutic effect on several neuropsychiatric-related diseases, such as hepatic encephalopathy, ASD and epilepsy (19–21). Previously, we found that FMT dramatically ameliorated the tic severity in one case (26). In this study, five patients who inadequately responded to medication intervention received one single FMT treatment. As the FMT procedures were variable, we chose to combine gastroscopy and colonoscopy under intravenous anesthesia for FMT treatment. Four of these patients exhibited clinical responses, with the exception of patient 2, indicating the significant efficacy of FMT in TS patients. All five patients showed good tolerance to the FMT treatment, and no one experienced any obvious adverse effects.

In our study, no statistically significant changes of LPS or cytokines in the five patients were observed. However, previous studies have shown that the peripheral immune system in patients with TS might be skewed to a pro-inflammatory state. LPS has been reported to significantly aggravate stereotypical and autonomic activity in TS rat models with increased IL-1β, IL-6, and TNFα levels as well as high expression levels of TLR4 (31). Here, we found that the LPS level in feces in patients 2 and 4 at baseline was higher than that in the donor, whereas it persistently decreased after FMT (W1, W8) and was consistent with the changes in serum, indicating that FMT might have reduced the pro-inflammatory immune responses of the above two patients. Previous studies reported that the pro-inflammatory cytokines IL-1β, IL-6, IL-12, IL-17, and TNFα are significantly elevated in patients with TS (12). The levels of IL-2, IL-12, and TNFα are positively correlated with the severity of tic symptoms (32).

In our study, the levels of IL-6 in patients 4 and 5, and IL-17F and IL-22 in patient 2 were down-regulated, suggesting a potential decrease in inflammation in these three patients. The impact of FMT treatment on the immune system cannot be ignored, while further studies with a larger sample size are needed to confirm the relevant alterations of LPS and cytokines after FMT.

Former literature focused on the composition and effect of gut microbiota in TS patients is rare. In our study, reduced gene numbers and a PCoA cluster separated from the donor cluster were observed at baseline, which is consistent with a previous study focusing on PANS/PANDAS showing a reduced operational taxonomic unit (OTU) number in the α-diversity analysis and a clear cluster apart from the healthy control cluster in the β-diversity analysis (33). Then, an obvious shift in the gut microbiota composition close to that of the donor microbiota was observed in four patients by the first week after FMT, indicating that the gut microbiota of the FMT recipients highly resembled that of the donor. However, the shift was transient, and the gut microbiota of the four patients deviated by week 8 to a state distinct from the baseline state. Interestingly, the tic symptoms of patients 1, 4, and 5 steadily improved by week 8, which is not consistent with the alterations in the overall microbiota shift. This phenomenon has also been observed in patients with other diseases treated with FMT, such as hepatic enteropathy (19) and refractory immune checkpoint inhibitor (ICI)-associated colitis (34). In addition, there is currently no uniform FMT frequency for the treatment of different diseases. Li et al. suggested that the interval should be <4 months for maintaining clinical efficacy in Crohn's disease after comprehensively assessing the changes in clinical symptoms, gut microbiota and metabolites (35).

In our study, changes in the abundance of three species remained stable at week 1 and week 8 after FMT: the relative abundance of *Bacteroides coprocola* and *Dialister succinatiphilus* was continuously increased in some of the patients, and *Bacteroides vulgatus* was absent in all patients. Moreover, *Bacteroides coprocola* abundance was negatively correlated with the improvement in tic symptoms. All the above results indicate that the three species have a potential influence on FMT treatment of TS. Previous studies reported that a special set of *B. coprocola* strains with a characteristic single nucleotide polymorphism (SNP) distribution was correlated with type 2 diabetes (T2D) (36). *D. succinatiphilus* participated in short-chain fatty acid (SCFA) generation via decarboxylating succinate to propionate (37). *B. vulgatus* is an opportunistic pathogen related to the increased incidence of T2D with increased inflammatory cytokines, specifically IL-6, and polycystic ovary syndrome by decreasing IL-22 secretion (38, 39), but negatively associated with atherosclerosis and LPS production (28). These findings suggest *B. vulgatus* plays an important role in different diseases through influencing the immune system. However, no obvious correlation between *B. vulgatus* and LPS/cytokines was observed in our study, likely due to the limitation of trial design and the sample size enrolled.

It is critical to explore alterations in metabolites related to the intestinal microbial compositions in TS patients receiving FMT. We found that after FMT, a number of metabolic pathways showed fluctuations, such as significant changes in amino acid metabolism, including the biosynthesis of arginine, lysine, valine, leucine, and isoleucine. Previous studies have revealed that dopamine, GABA and glutamate, as neurotransmitters and the metabolites of amino acids, greatly influence tic pathophysiology (40); furthermore, GABA and glutamate antagonists have been suggested as treatment options for TS (41). However, the literature focusing on the microbiota and metabolites in the gut of patients with TS is scarce and more studies are urgently required.

This pilot study reported the efficacy and safety of FMT on five TS patients and the specific alterations of fecal microbial composition and serum cytokines following FMT. However, there are several limitations. Only a small number of patients was enrolled without a control group. The follow-up period is relatively short and long-term effect of FMT on TS cannot be estimated. Nevertheless, our study indicated the crucial role of gut microbiota in the pathogenesis of TS. Together, this study provides novel evidence that reconstitution of the gut microbiota through FMT might be a safe and effective alternative therapy for TS. A large-scale randomized, controlled clinical trial with a longer follow-up is needed to confirm the efficacy and safety.

## Data Availability Statement

The datasets presented in this study can be found in online repositories. The names of the repository/repositories and accession number(s) can be found below: NCBI BioProject (accession: PRJNA628029).

## Ethics Statement

The studies involving human participants were reviewed and approved by the Ethical Committee of Chinese PLA General Hospital. Written informed consent to participate in this study was provided by the participants' legal guardian/next of kin.

## Author Contributions

H-JZ and XL collected the samples, analyzed the data, and drafted the manuscript. Y-CY, L-HP, Y-CS, and J-FL performed the FMT treatments. GY, JW, and L-PZ enrolled the eligible patients. X-YS and L-YH conducted the YGTSS-TTS. FP and R-RR assisted with analysis. Y-SY and L-PZ designed, funded, revised manuscript, and supervised the study.

## Conflict of Interest

The authors declare that the research was conducted in the absence of any commercial or financial relationships that could be construed as a potential conflict of interest.
