# Supplementary figures and images for: The Efficacy of Fecal Microbiota Transplantation for Children With Tourette Syndrome: A Preliminary Study
Source: Front Psychiatry. 2020 Dec 23;11:554441. doi: 10.3389/fpsyt.2020.554441 (PMC7793740; doi:10.3389/fpsyt.2020.554441)

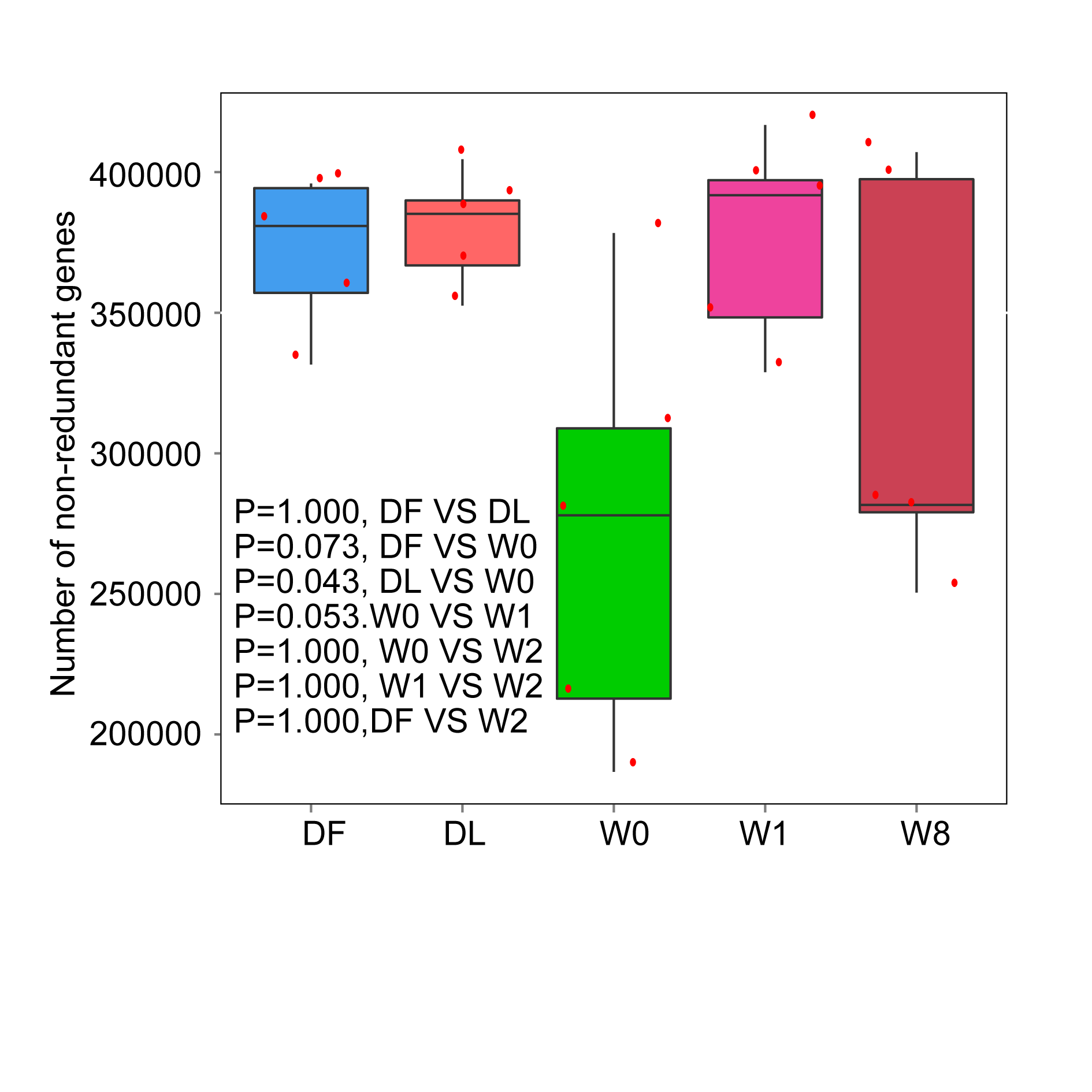

Supplement: Supplementary file 1 [file Image_1.TIF]

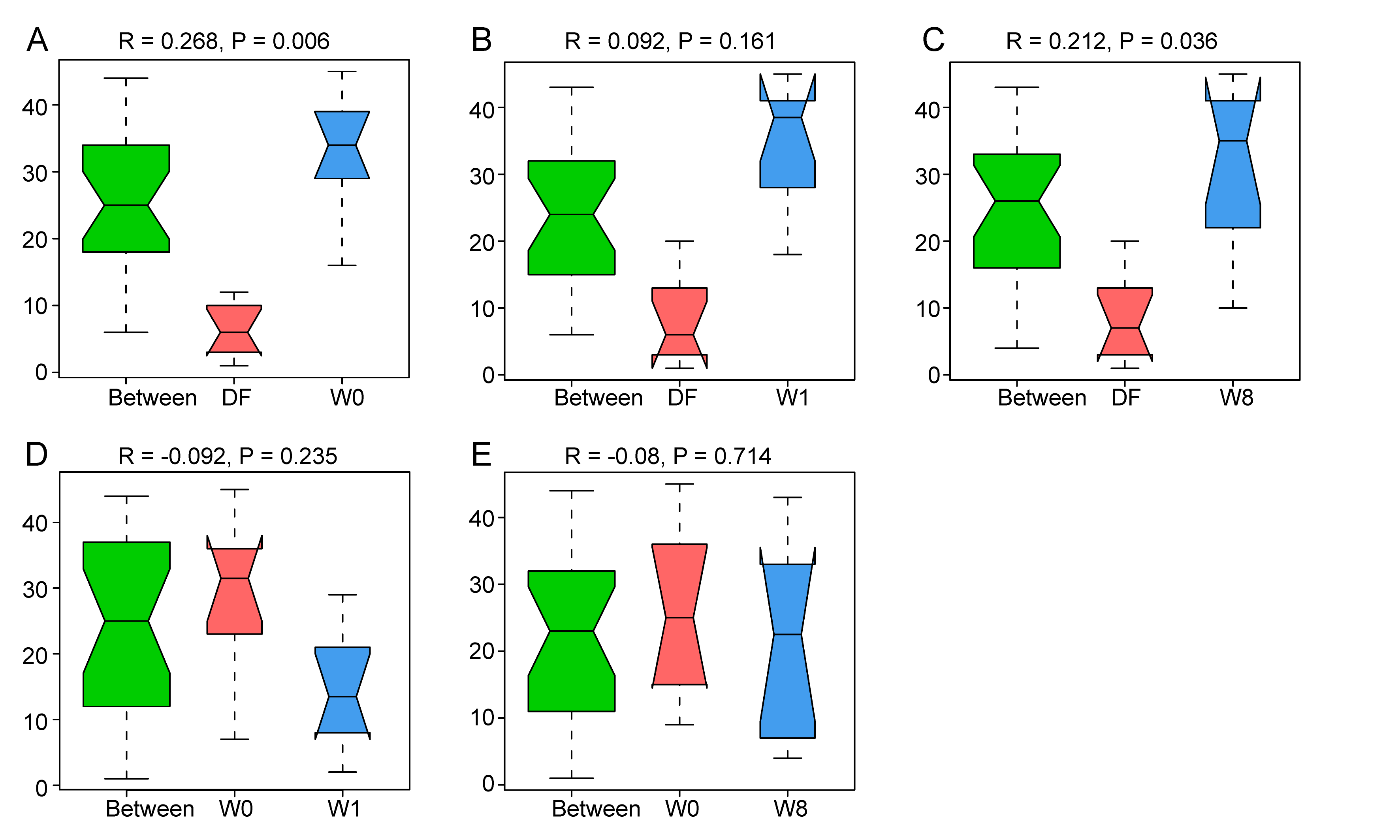

Supplement: Supplementary file 2 [file Image_2.TIF]

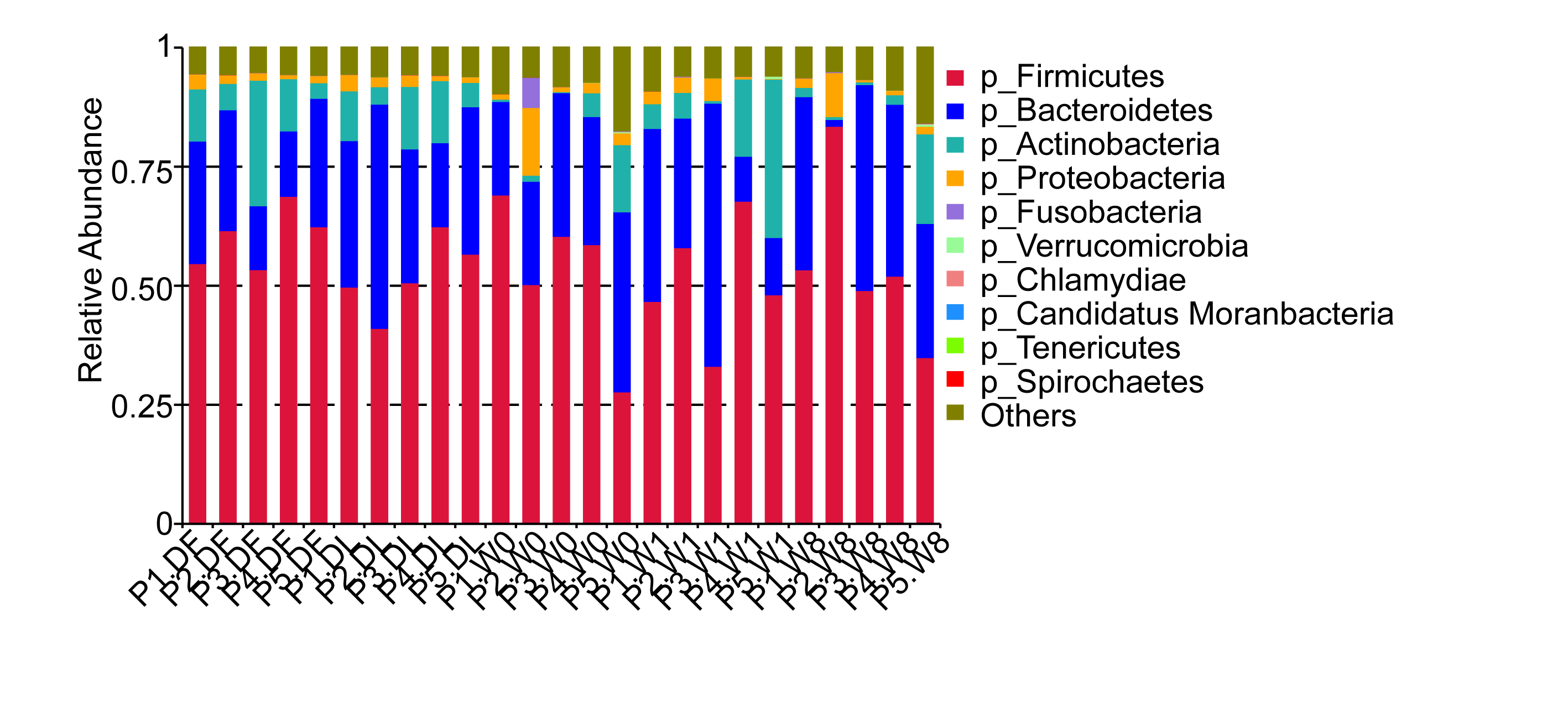

Supplement: Supplementary file 3 [file Image_3.TIF]

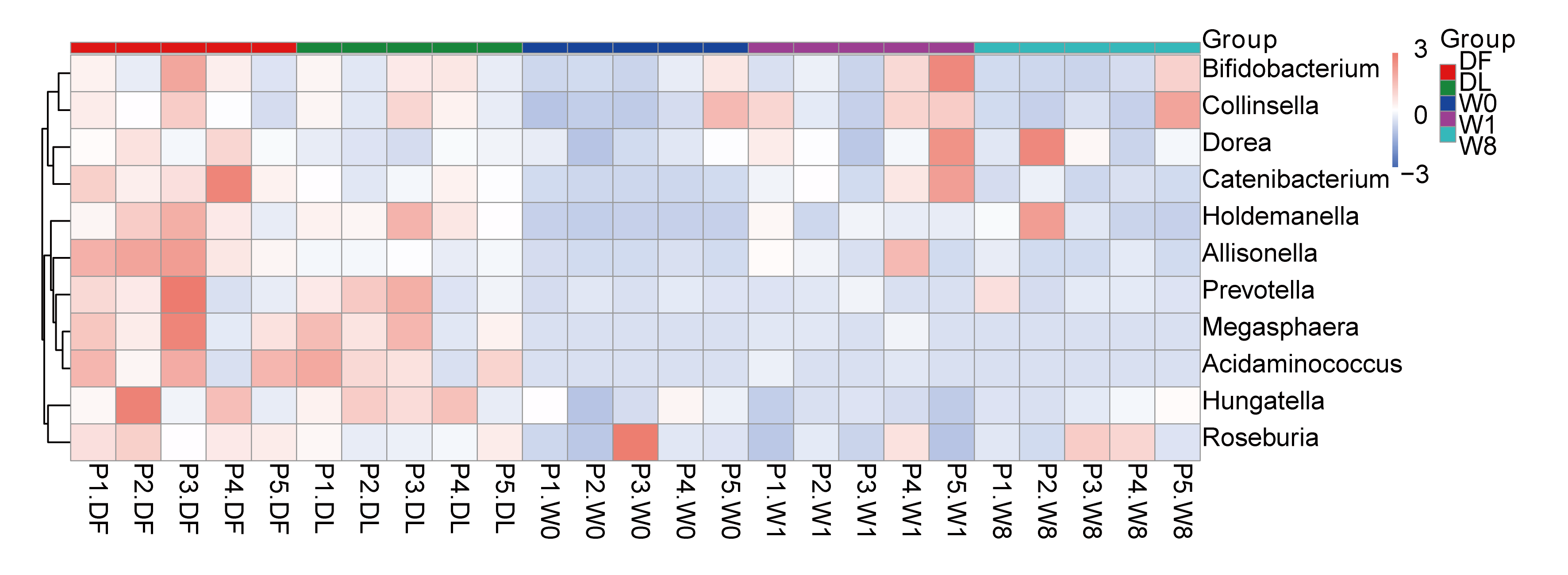

Supplement: Supplementary file 4 [file Image_4.TIF]
