# Supplementary material for: The Efficacy of Fecal Microbiota Transplantation for Children With Tourette Syndrome: A Preliminary Study
Source: Front Psychiatry. 2020 Dec 23;11:554441. doi: 10.3389/fpsyt.2020.554441 (PMC7793740; doi:10.3389/fpsyt.2020.554441)
Supplement: Supplementary file 5 [file Data_Sheet_1.docx]

**Supplemental material**

**Figure S1** The non-redundant gene count of faecal samples was compared. The P value is from the one-way ANOVA with Tukey’s post hoc test (DF: donor faeces; DL: donor faecal liquid; W0: baseline of patients; W1: week 1 after FMT; W8: week after FMT).

**Figure S2** ANOSIM showing a significant difference in DF VS W0, W0 vs W1, and DF VS W2 (P<0.05), but no significant difference in W0 VS W2, or DF VS W1 (P>0.05), indicating different microbial communities between TS patients and healthy donors. After FMT treatment, the microbial composition was transiently similar to that of the donor (W1) but later returned to the original state. (DF: donor faeces; DL: donor faecal liquid; W0: baseline of patients; W1: week 1 after FMT; W8: week after FMT).

**Figure S3** Relative abundance of the top 10 microbes at the phylum level per sample. (DF: donor faeces; DL: donor faecal liquid; W0: baseline of patients; W1: week 1 after FMT; W8: week after FMT).

**Figure S4** Changes in the relative abundance of 11 genera after FMT generated with LEfSe. (DF: donor faeces; DL: donor faecal liquid; W0: baseline of patients; W1: week 1 after FMT; W8: week after FMT).
